# The role of patient organizations in the rare disease ecosystem in India: an interview based study

**DOI:** 10.1186/s13023-019-1093-6

**Published:** 2019-05-29

**Authors:** Mohua Chakraborty Choudhury, Gayatri Saberwal

**Affiliations:** Institute of Bioinformatics and Applied Biotechnology, Biotech Park, Electronics City Phase 1, Bengaluru, Karnataka 560100 India

**Keywords:** Rare disease, Patient group, Disease-specific group, Umbrella organization, Policy

## Abstract

**Background:**

Rare diseases (RDs) affect a small percentage of the population but can be severely debilitating and life-threatening. Historically, patient groups (PGs) have been the prime movers in raising awareness about these diseases and advocating for national supportive policies. They have also driven relevant research programs. In India too, PGs have made significant contributions to the national RD ecosystem.

**Objective:**

To assess the contribution of various Indian RD PGs, we carried out an interview-based study of 19organizations. This study aims to highlight the origins and achievements of these groups and the challenges that they have faced. The study also helps to capture the changes in the RD ecosystem that have taken place in recent years.

**Result:**

Of the 19 PGs, two are umbrella organizations, two are other organizations of national scope and 15 are disease specific groups. 14 interviewees were affected by an RD either directly or through a family member. Lack of awareness about RDs in the medical community was the biggest challenge, leading to a delay in diagnosis and subsequent management. Only two out of the 15 conditions have a definitive treatment. However, many of the diseases can be managed with replacement therapies administered for the patient’s entire life, or other supportive therapies. Most diagnostics and treatment regimens that are available globally are now available in India but are expensive and usually available only in a few major cities. These problems are compounded by a lack of medical insurance schemes and government policies to support these patients. The biggest achievement of the PGs was the passing of National Policy for Treatment of Rare Diseases in 2017, unfortunately since put in abeyance. If reinstated, and properly implemented, this policy could significantly improve RD management in the country.

**Conclusion:**

PGs have had a significant role in bringing diagnostics and treatments to India. They have also raised awareness about RDs and related issues such as newborn screening, prenatal diagnostics and genetic counselling. This study highlighted the recommendations of various PGs. The government should address these recommendations and institutionalize the participation of the PGs in formal decision making.

**Electronic supplementary material:**

The online version of this article (10.1186/s13023-019-1093-6) contains supplementary material, which is available to authorized users.

## Background

A rare disease (RD) affects a small percentage of the population. However, there is no universal definition for an RD and each country has adopted a definition based on its population, healthcare system and resources. Disease prevalence is the most widely used epidemiological metric: 28 countries of the European Union (EU) use the figure of five per 10,000 whereas the United States of America (US) and Japan define an RD as one that, at any given point in time, affects less than 200,000 and 50,000 patients respectively [1]. Some definitions also include other criteria such as disease severity and whether it is genetic in nature, and this has resulted in as many as 296 definitions for an RD [2]. Collectively, RDs affect a large population, and pose a significant challenge to the public health system of any country [3]. They can also have a devastating effect on the lives of patients and their caregivers [4].

The first published reference to a rare disease in India was in 1967 [5]. However, the reporting of cases is poor, and so far only about 450 RDs have been recorded [6]. Even as recently as the early 2000s, the medical fraternity, the government and the general public were largely unaware of these diseases, which have rarely figured on the medical or scientific agendas of the country. Although there has been no systematic prevalence study, extrapolating from international data, it is estimated that there are 72 to 96 million RD patients [6]. To be noted is that due to the wide diversity in the Indian population, and both endogamy and consanguinity in many ethnic groups, some diseases might be more prevalent in certain groups or regions [7, 8]. Medical genetics and rare disorders have not been given importance in the medical college curriculum [7], and as a result most cases remain undiagnosed. For the cases that have been correctly identified, the diagnosis was usually made at a tertiary hospital [9, 10]. Further, the exorbitant prices of orphan drugs, compounded by the widespread lack of health insurance makes even the available RD treatment inaccessible for most patients [11, 12]. The cost of drugs such as Spyrine and Venclext approaches Rs. 0.5 million for a year’s treatment, which exceeds most Indians’ annual income. Since most of these treatments are lifelong, treatment costs can mount to tens of millions of rupees per patient over time [13].

Globally, patient groups (PGs) have played a huge role in shaping the agenda for the care of RD patients. The historic Orphan Drug Act of 1983 in the US resulted from a movement that was largely led by PGs [14]. This Act inspired similar laws in many other countries, such as Japan (in 1993), Australia (1998) and the EU (2000) [15, 16]. Aside from garnering the attention of policy makers, patient involvement has helped to draw the attention of the medical community, the scientific community and industry to RDs [17, 18]. PGs are also important sources of information for patients and caregivers on what is available in terms of diagnosis, management and treatment of the condition or disease [19]. Further, there are now many examples of PGs that have initiated or supported significant research programs [20–24] and some of these have led to the development of new treatment for diseases such as cystic fibrosis [25], lymphangiomyomatosis [26] and Pompe’s disease [27].

Vigorous advocacy by several PGs has raised the profile of RDs in India in recent years. Nevertheless, and despite many achievements, the resources available to local RD patients are still very limited. It is therefore important to highlight and analyze the efforts of the PGs to date. Although this has been done in some other countries [28–30], a comprehensive assessment of the role of such organizations in India has not been carried out so far. We therefore undertook an interview-based study to analyze the origin and activities of some of the major PGs in India. We captured how these groups originated and evolved, what major challenges they faced, and how their efforts improved the ecosystem for these patients. This study also helped us identify areas which need the immediate attention of the government and other stake-holders.

### Methodology

We adopted a qualitative case study research methodology, using semi-structured interviews. A questionnaire (Additional file 1) was used to elicit detailed accounts from the PGs.

### Ethics, consent and permission

The study was approved by the Institutional Ethics Committee of the Institute of Bioinformatics and Applied Biotechnology, Bangalore. To indicate their consent to participate in the interviews, potential interviewees were asked to respond positively to an interview invitation sent by e-mail, and to sign an informed consent form.

Through an information sheet, all the interviewees were informed about (i) the affiliation of the researchers, (ii) the source of funding for the project, (iii) the objectives of the study, and (iv) the investigators’ intention to publish the findings in an academic journal without seeking their approval of the manuscript. Although participants were assured that their responses would be kept confidential, subsequently, we obtained permission to use particular information from the interviews in the intended publication. Finally, participants were informed that they would not be paid for participating in the study.

### Data collection

We chose the snowballing method to choose RD PGs. We set out to classify the organizations into two broad categories, disease-specific groups (DSGs) and umbrella organizations (UOs). The inclusion criteria for each of these categories are listed below.

For DSGs, the organization should:

i. represent the interests of patients with a particular RD
ii. have substantial involvement with patients
iii. be engaged in informing and supporting patients

We did not consider registration status as an inclusion criteria as some of the PGs such as MERD-India and Sjögren’s India were not registered at the time of the study although, these organizations have had substantial impact in their disease area.

For UOs, the organization should:

i. represent the interest of all RD patients
ii. have been setup with primary focus on RDs
iii. be national in scope
iv. cover two or more issues of concern to all patients with RDs in India
v. be involved in activism and advocacy on behalf of RD patients both at national and international level
vi. have good visibility among the RD patients, DSGs and international RD fora

However, there were two other organizations whose interests covered all RDs but which did not satisfy some of the criteria used to define a UO. We have therefore classified these two organizations as Other Organizations of National Scope (OONS). We purposively selected 19 well-known RD patient organizations. Of these, only two could be classified as UOs: Indian Organization for Rare Diseases (I-ORD); and Organizations for Rare Diseases India (ORDI). Two organizations, Centre for Health Ecologies and Technology (CHET) and Foundation for Research on Rare Diseases and Disorders (FRRDD), were classified as OONS. The remaining 15 were classified as DSGs: Asha Ek Hope Foundation (AEHF); Down Syndrome Federation of India (DSFI); Dystrophy Annihilation Research Trust (DART); Fragile X Society - India (FXS-I); Hemophilia Federation of India (HFI); Iksha Foundation (Iksha); Indian Institute of Cerebral Palsy (IICP); Indian Patients Society for Primary Immunodeficiency (IPSPI); Indian Rett Syndrome Foundation (IRSF); Lysosomal Storage Disorder Support Society (LSDSS); Metabolic Errors and Rare Disease Organization of India (MERD India); Multiple Sclerosis Society of India (MSSI); Sjögren’s India (SI); Thalassemia and Sickle Cell Society (TSCS); and World Without GNE Myopathy (WWGM). In addition, we interviewed one patient-activist, Ms. Dhanya Ravi, who did not represent any PG.

The questionnaire was refined by conducting five pilot interviews, which helped to refine it prior to the implementation of the study. Only two interviewees were known to the study team prior to the start of the study. For each interview, a key person in the organization, who was usually a founder, was first contacted by email, and a preparatory call was set up to brief him or her on the objective of the study, to ascertain interest in participating in the study, and to schedule the interview. We conducted the interviews in a semi-restrictive manner so as to give the interviewees space to discuss their story in detail and raise issues related to the RD of interest. For those located in Bangalore, the interviews were conducted face-to-face, at a location convenient to the interviewee, usually his or her office or home. For those located elsewhere, the interviews were conducted by phone or skype. The interviews lasted between 90 and 180 min and on average took 105 min. The longer interviews were conducted over two or three sessions according to each interviewee’s convenience. All interviews were conducted by MCC between June–September 2018, inclusive. The interviews were audio recorded and transcribed verbatim for the purpose of analysis. Occasionally, the interviewers were directed to the organization’s informational materials or published interviews for further information. Also, some interviewees were subsequently contacted for clarifications.

### Data analysis

The transcripts were analyzed using thematic analysis. The questionnaire was divided into five major sections, as follows: (i) details of the PG; (ii) details of the disease and key patient around whom the PG was formed; (iii) challenges faced by the PG; (iv) achievements of the PG; and (v) the PG’s recommendations to the government. Each interview transcript was shared with the interviewee for his or her feedback. In order to use some parts of the interview in the manuscript with attribution, relevant extracts of the manuscript were subsequently shared with the interviewee to obtain explicit consent for this. However, the final manuscript was not shared with any interviewee for review. Although the interviewees are identified in Table 1, as far as possible we have used organizations’ names instead of interviewees’ names in the paper. Also, although some of the RDs may be conditions, and not diseases, for convenience we refer to all of them as diseases. The affected individuals are referred to as patients.

**Table 1:** Details of the PGs and interviewees

	Organization details			Interviewee details		
	Organization name *Official website*	Disease of interest	Location of headquarters, Year of registration, if applicable [Year of start of work]	Name of the interviewee	Is the interviewee a founding member?	Interviewee involved with an RD in what capacity?
	UMBRELLA ORGANIZATIONS (UOs)					
1	Indian Organization for Rare Diseases (I-ORD) *http://www.i-ord.org/*	All rare diseases	In US: Minnesota, 2005; In India: Hyderabad, 2008 [2005]	Dr. Ramaiah Muthyala	Yes	Volunteer/ Scientist
2	Organizations for Rare Diseases India (ORDI) *https://ordindia.org/*	All rare diseases	In India: Bangalore, 2014; In US: Virginia, 2017 [2004]	Mr. Prasanna Shirol	Yes	Parent
	OTHER ORGANIZATIONS OF NATIONAL SCOPE (OONS)					
3	Centre for Health Ecologies and Technology (CHET) *http://iiacd.org/chet.html*	All rare diseases	Bangalore, 2015	Dr. Namitha A. Kumar	Yes	Patient
4	Foundation for Research on Rare Diseases and Disorders (FRRDD) *http://www.rarediseasesindia.org/*	All rare diseases	Chennai, 2010 [2009]	Dr. Duraiswamy Navaneetham	Yes	Volunteer/ Scientist
	DISEASE SPECIFIC GROUPS (DSGs)					
5	Asha Ek Hope Foundation (AEHF) *http://www.ashaekhope.com/*	Amyotrophic lateral sclerosis (ALS)	Mumbai, 2011 [2004]	Ms. Shera Mukherjee	No	Patient
6	Down Syndrome Federation of India (DSFI) *http://www.downsyndrome.in/*	Down syndrome (DS)	Chennai, 1984	Dr. Surekha Ramachandran	Yes	Parent
7	Dystrophy Annihilation Research Trust (DART) *https://dartindia.in/*	Muscular dystrophy (MD)	Bangalore, 2012	Mr. Ravdeep Singh Anand	Yes	Parent
8	Fragile X Society - India (FXS-I) *http://www.fragilex.in/*	Fragile X syndrome	Mumbai, 2007	Ms. Shalini Kedia	Yes	Parent
9	Hemophilia Federation of India (HFI) *http://hemophilia.in/*	Hemophilia	Delhi, 1983	Mr. Vikash Goyal	No	Parent
10	Iksha Foundation (Iksha) *http://ikshafoundation.org/*	Retinoblastoma	Bangalore, 2010	Mr. Thanmaya Bekkalale	Yes	Volunteer
11	Indian Institute of Cerebral Palsy (IICP) *http://www.iicpindia.org/*	Cerebral palsy (CP)	Kolkata, 1974	Dr. Sudha Kaul	Yes	Parent
12	Indian Patients Society for Primary Immunodeficiency (IPSPI) *https://www.ipspiindia.org/*	Primary immune deficiency (PID)	Noida, 2004	Ms. Rubby Chawla	Yes	Parent
13	Indian Rett Syndrome Foundation (IRSF) *http://www.rettsyndrome.in/*	Rett syndrome (RS)	Delhi, 2010 [2007]	Dr. Rajni Khajuria	Yes	Volunteer/ Scientist
14	Lysosomal Storage Disorder Support Society (LSDSS) *http://www.lsdss.org/*	Lysosomal storage disorder (LSD)	Delhi, 2010	Mr. Manjit Singh	Yes	Parent
15	Metabolic Errors and Rare Disease Organization of India (MERD India) *http://www.merdindia.com/*	Inborn errors of metabolism (IEM)	Jaipur, not registered [2011]	Mr. Vikas Bhatia	Yes	Parent
16	Multiple Sclerosis Society of India (MSSI) *http://www.mssocietyindia.org*	Multiple sclerosis (MS)	Delhi, 1985	Ms. Renuka Malakar	No	Spouse
17	Sjögren’s India (SI) *http://www.sjogrensindia.org/*	Sjorgen’s syndrome (SS)	Ahmedabad, not registered [2006]	Ms. Kirtida Oza	Yes	Patient
18	Thalassemia and Sickle Cell Society (TSCS) *http://tscsindia.org/*	Thalassemia and sickle cell anemia	Hyderabad, 1998	Dr. Suman Jain	Yes	Volunteer
19	World Without GNE Myopathy (WWGM) *http://gne-myopathy.org/*	GNE myopathy (GM)	Delhi, 2015	Dr. Shilpi Bhattacharya	Yes	Patient

## Results

Twenty-one PGs were invited to participate in the study, of which 19 agreed. Basic information about the 19 interviewees and their organizations is available in Table 1. As mentioned above, of the 19 PGs, four organizations represent the interests of all RDs, of which two are UOs and two are OONS. The remaining 15 are DSGs which focus on one disease each. Of the 15 DSGs, 11 relate to an early-onset disease and four to a late-onset one.

The following 12 themes were used to carry out a thematic analysis of the interview transcripts: How interviewees got involved with an RD, prevalence, PGs’ outreach to patients, diagnosis, treatment, management, schooling, counselling, patient registries, research, achievements and recommendations.

### How interviewees got involved with an RD

Among the 19 interviewees, 14 were affected by an RD either directly or through a family member. Four were patients, 9 were parents and one had an affected spouse (Table 1).

The common driving force for each person to create or join a PG was the lack of a supportive ecosystem. All of the interviewees who had had to deal with an RD had faced many challenges. Lack of awareness about RDs in the medical fraternity was the biggest challenge, leading to a delayed diagnosis for most patients. Further, there was no central source of information about the RD. All of this made the patients and caregivers feel helpless. Ms. Dhanya Ravi, who was diagnosed with Osteogenesis imperfecta (OI) 52 day after birth, grew up thinking she was the lone survivor with this condition in the country. It was only at the age of 18 that she got to know about other OI patients when she chanced upon a fund-raising advertisement in a newspaper by another OI patient.

Eleven of the 15 DSGs were the first for that disease in India whereas the other four interviewees joined existing DSGs (Additional file 2). Some of the founders were inspired by PGs in other countries and took the initiative to start one in India. Five of the 19 organizations were set up by individuals who were not personally affected by an RD. In four cases the founders were scientists who got involved through their work. Some of the founders of FRRDD, I-ORD and ORDI were Indian scientists based in the US who were inspired by the RD movement there. IRSF was also founded by a scientist. TSCS was founded by medical doctors who had patients with rare blood disorders, and Iksha was started by a couple who got involved with retinoblastoma after their son was incorrectly diagnosed with it.

### Prevalence

Since a nationwide prevalence study has not been carried out for any of the diseases considered here, some academic research groups and DSGs have taken the initiative to carry out such studies on their own. A multicenter study, covering cities from different regions of the country, has been carried out to screen for β-thalassemia carriers and other haemoglobinopathies. This has revealed a prevalence of 0—10.5% among different caste and ethnic groups [31]. Although GNE myopathy (GM) has a prevalence of six per million internationally, a study conducted by WWGM found that the relevant mutation is common in certain ethnic groups in India, resulting in a large carrier population in these groups [32]. Therefore, GM is expected to have a higher prevalence in the country than elsewhere. As such, prevalence figures for any RD in India might be incorrect or even completely misleading. DART is beginning its own study with the help of the state government. Known as the ‘KIDS program: Karnataka Intensive Dystrophy Survey’, the objective is to understand the prevalence of muscular dystrophies in the state of Karnataka and raise awareness in every district. Muscular dystrophy patients will be identified by trained personnel in the Primary Health Centers (PHCs).

### PGs’ outreach to patients

All the UOs and OONS support patients, who reach out to them by connecting them to the relevant DSGs. For most DSGs, reaching out to patients or their families was initially difficult. Illustratively, Mr. Manjit Singh of LSDSS used to visit the pediatric outpatient department (OPD) of various hospitals in search of Lysosomal Storage Disorder (LSD) patients, to persuade them to join the group. In this manner he found about 10 patients with whom the LSDSS group was set up. Most organizations were started in a similar manner, by one or two families taking the initiative and contacting other families to form a small group.

From the beginning, all the PGs have created awareness about their RD of interest through talks at conferences and seminars. This gave the groups visibility in the medical community, and doctors then referred patients to them. In this way informal networks with the medical community emerged, and the PGs engaged with more patients over time. Of late, patients have learned about them through web searches and through the media.

Many of the PGs also network with groups such as those of Accredited Social Health Activists (ASHAs), who are trained to work as an interface between the community and the public health system, and those at the Anganwadi centers, which are child care centers primarily in rural areas. Underprivileged families, who find it difficult to access formal health services are often in touch with these resource people. In several places these personnel have been trained to identify the early symptoms of certain conditions and to urge the affected families to seek medical help. They can also identify families with a high incidence of a particular RD and counsel them on the possibility of monitoring future pregnancies.

### Diagnosis

All the 14 interviewees who were affected by an RD directly or through a family member faced a delayed diagnosis. Very few primary care physicians are aware of the relevant symptoms and there are also very few specialty centers in the country. Even for diseases with prominent symptoms it can take many years to diagnose the condition. Illustratively, Mr. Shirol mentioned that it took seven years to diagnose his daughter with Pompe disease although there were symptoms since the child was few months old.

In the case of the late onset diseases – GM, multiple sclerosis (MS), and Sjögren’s syndrome (SS) – most patients had an almost normal life before the symptoms appeared. Mrs. Malakar’s husband served as a fighter pilot in the Indian Air Force and was absolutely physically fit until the appearance of symptoms of MS. It took five years from the occurrence of major symptoms to diagnosis. Kirtida Oza of SI narrated her saga: “From my late 20’s, I suffered from multiple problems and visited various specialists who treated me like a ‘bag of organs’, each focusing on one part, but failing to recognize the systemic nature of the problem. Frustrated, I researched possible causes and stumbled upon an article on SS. I was convinced that I had it. Since there were no rheumatologists in my city at that time, I sought an appointment with one in Mumbai. After a detailed case history, specific diagnostic tests, and a procedure he finally confirmed my suspicion. It took around 5 years to get a proper diagnosis of primary SS.”

The non-availability of diagnostic tools and techniques is another area of serious concern. The interviewees pointed out that even common diagnostics such as karyotyping for Down’s Syndrome (DS), enzyme assembly tests for LSD, and partial thromboplastin time and activated partial thromboplastin time for hemophilia are not commonly available outside a few major cities. Genetic testing too is largely unavailable or unaffordable.

Newborn screening can aid in the early diagnosis of most early-onset diseases such as blood disorders, inborn errors of metabolism (IEM), LSD, Primary Immune Deficiency (PID) and the muscular dystrophies. MERD-India mentioned that “a child with an IEM can survive only if its condition is identified immediately after birth. If the baby is symptomatic and is found to be positive during screening it has to be put on a special diet at once, while waiting for a confirmed diagnosis.” Therefore, MERD India has relentlessly pushed the government to make newborn screening mandatory. It has raised the issue at various fora such as the National Neurology Forum, and with the committee drafting the national policy for RDs in India. Owing to all these campaigns, newborn screening has been made available by the governments of Kerala and Tamil Nadu, and in hospitals elsewhere such as the Cloud 9 chain of hospitals; Manipal Hospital, Mangalore; and the Sanjay Gandhi Postgraduate Institute of Medical Sciences, Lucknow.

Prenatal diagnosis is possible for 10 diseases in our cohort, and all the concerned DSGs strongly advocate it (Additional file 2). Usually such screening is recommended if the fetus displays an abnormal phenotype or if the family has a known risk. The parents of an affected child are made aware of the genetic nature of the disease, and doctors and counsellors at the hospital advise them to monitor future pregnancies. DSGs, such as HFI, Iksha and TSCS also provide financial support for pre-natal diagnosis. TSCS has been a pioneer in establishing prenatal screening programs for thalassemia in the states of Andhra Pradesh and Telangana. These programs focus on carrier testing, pre-marriage counselling of close relatives, and prenatal testing for subsequent pregnancies. Cerebral palsy (CP), which does not have a genetic link, can also benefit from prenatal screening and monitoring. IICP works closely with clinics where they monitor the pregnancies of women who have had tuberculous meningitis, a leading cause of neurological disorders. A patient with SS is often diagnosed only after she has had multiple miscarriages. She is closely monitored during pregnancy, since the child may develop complications such as a heart block.

### Treatment

Most RDs do not have a cure (Additional file 2). Thalassemia and retinoblastoma are curable, but only if diagnosed early, and bone marrow transplantation (BMT) for thalassemia has to be done between the ages of two and eight. The primary care physician is often unable to make a timely diagnosis or initiate management of the disease. Also, both BMT and retinoblastoma eye surgeries are expensive, and carried out at only a few centers in the country. Therefore, most patients are unable to access or afford them.

### Management

The management of most RDs has become easier over the decades. Mrs. Rubby Chawla of IPSPI mentioned that in the early 1990s Sandoz’s Intravenous Immunoglobulin (IVIG), was very expensive and had to be imported. Later, Indian companies started marketing affordable products from China and Korea. Now IVIG is manufactured locally. This has reduced the cost further, and availability is no more a challenge. DSFI mentioned that in the 1980s, post-operative care of persons with DS was very poor, and many children suffered complications and could not recover properly. Since then, post-operative care has significantly improved.

Devices used to manage some RDs have recently become available. Examples include a customized wheelchair for muscular dystrophies and assistive communicative devices for CP. However most of them are not produced locally and are very expensive. Other lifesaving products such as IEM diets, enzyme replacement therapies, IVIGs and blood factors are also expensive and only available at a few specialized centers. This makes them inaccessible to most patients. For many years, MERD India lobbied the Food Safety and Standards Authority of India (FSSAI) to bring special diets to India. This led to the FSSAI’s ‘Diet for Life’ project which was geared to making IEM diets readily available in India. As a result, five multinational companies obtained marketing rights for their diets, and the local firm Pristine Organics also started to manufacture such diets. These special diets are now readily available and can be purchased online. However, there is still a dearth of nutritionists who can provide guidance on the specific requirements of a given patient.

Most of the interviewees felt that tertiary interventions, which include specialized physical and mental exercises, are very important. Such programs have to be customized for each child, since each has different mental and physical abilities, and medical needs. Various therapeutic activities such as art, music, drama and sports can also significantly impact a child’s development, especially if taken up early and tailored to his or her interests. However by and large such management has not been prioritized, and the country still lacks the relevant expertise to run such programs. DSFI noted that early interventions are very important for children with DS, where they focus on the positioning of infants – that is, seating, carrying, laying down and so on – to encourage normal postural patterns. This PG instructs parents on how to teach their babies through daily activities like feeding, changing nappies and bathing. It is also very important to adopt an integrated approach towards managing a disease. TSCS has specialized dentists, endocrinologists, gastroenterologists and other doctors who are well aware of possible complications, and provide customized care to thalassemic patients.

Three of the PG founders have earned a professional degree in the management of their disease of interest and are developing management methods customized for Indian conditions (Additional file 2).

### Schooling

Children with RDs can vary greatly in their intelligence and normalcy of behavior, and their educational needs. The educational system is by and large not geared to meeting these needs, and in many cases schools have been unsupportive. Most DSGs work to sensitize school managements and make them more accommodative of these needs. DSFI, IPSPI and other PGs reach out to schools when parents report a problem. IICP works with government schools in Kolkata to ensure that these students are accommodated. Organization such as Iksha also help finance the education of patients.

With the efforts of the DSGs, the situation has improved. Mr. Anand’s son is affected by DMD, and his classroom was on the ground floor for many years. When it was shifted to a higher floor, a ramp was constructed. The lobby, washrooms and even laboratories were made wheelchair friendly. Also, efforts were made to sensitize other children to his condition.

In general, smaller schools are more accommodating. Most PGs agreed that special schools would not help, as a city might have only a few such schools and they would not be accessible to many children due to distance. Furthermore, a special school would limit a child’s developmental targets. So it is essential that these children attend normal schools which have special educators, shadow teachers and resource rooms with special aids. However, it is very challenging to accommodate children with severe intellectual or physical disabilities. Ms. Dhanya Ravi could not attend school due to the constraints imposed by OI, but with proper home schooling she has built a successful career as a content writer and an RD patient activist.

### Counselling

RDs cause patients and their families a lot of stress. To quote DART “It usually starts with denial, and then depression which can last from months to years for different families. One of the parents has to stop working and stay home to take care of the child while the other is also unable to give full attention at work. Health expenses increase while income decreases.” CHET recalled a person in his early twenties who committed suicide as he was unable to bear the progression of juvenile-onset Huntington’s disease. He was in Mumbai and had access to the best hospitals but a supportive ecosystem did not exist.

Fifteen of the 19 PGs counsel patients and provide moral support (Additional file 2). IPSPI provides counselling on the genetics of the disease, its psychological impact, the importance of IVIG and monitoring future pregnancies. MERD India makes families aware of newborn screening, accurate diagnosis and a special diet. IICP works with government hospitals and also hosts an OPD where it provides counselling and training to parents, and acts as an early intervention center. Several diseases, such as hemophilia, thalassemia, LSD and the PIDs require replacement therapies, and families have to be counselled on the risks associated with missing regular medication. Many of the DSGs are active on social media thereby enabling people from different parts of the country to interact and share knowledge. It can be very challenging to educate the patient and his or her family, especially if they do not access the electronic media. Organizations such as DSFI, Iksha, IPSPI and TSCS produce informational booklets and posters in local languages. This is helpful to people who do not use the internet or are comfortable only in a certain language. As mentioned earlier, several PGs counsel families during pregnancy in particular, and recommend early screening for RDs.

### Patient registries

Ten of the 19 PGs maintain patient registries (Additional file 2). These databases list patients who are associated with the PG, or who had contacted the organization for help at some point. Organizations such as TSCS also maintain the medical history of each registered patient (Additional file 2). TSCS has records of 21,500 patients followed by MSSI and HFI with 3500 and 2486 respectively. Although these are large numbers, they are just a small sample of the patients in the country, and none of the registries is comprehensive.

ORDI has launched a national helpline phone number + 918,892 555,000 since 2014. So far more than 5000 patients have contacted and their information is stored in a registry. FRRDD aims to identify existing registries scattered around the country and coordinate with the relevant PGs to develop a comprehensive online RD registry. However, DART and LSDSS would prefer to contribute to a national registry rather than run their own. It is therefore very important to have a comprehensive national RD registry, such as that suggested in the NPTRD (http://bmi.icmr.org.in/irdr/index.php), which is regularly updated.

### Research

Thirteen organizations (Additional file 2) felt that research is important to find either a cure or an intervention for better disease management. Some of them are involved in research, either independently or in collaboration. Illustratively, WWGM carried out a study to investigate the mutation spectrum of GM in India; the founder of IRSF has enabled the molecular diagnosis of Rett syndrome by identifying the mutation spectrum of Indian patients; DART is carrying out a genetic study which will lead to the development of personalized genomic interventions; DSFI is working on nutrition and the benefits of aqua-therapy for DS; and MSSI plans to conduct a study of the benefits of yoga for MS patients. TSCS aims to understand the observed phenotype and correlate it with the underlying genotype by understanding the primary, secondary and tertiary genetic modifiers. It has been found that of the 200 mutations known globally, 28 mutations occur very frequently in India, and about 90% of the patients of their study have one of six mutations [31].

Aside from its medical research, IICP’s research on Augmentative and Alternative Communication devices and aids has resulted in many improved teaching and learning methods for CP patients [33]. For instance, it has worked to develop a video-based training module for community-based rehabilitation workers in rural India. It has also partnered with Dimagi, a technology company that specializes in providing mobile technology to underserved communities across the globe, to spread the use of its training modules.

Some of the PGs believe that the government should work to make India a hub of RD research and treatment. A major challenge in such research is the small number of patients in most parts of the world, but for many RDs the number of patients in India is large simply because of the country’s large population [34]. Were such research to be ramped up, it would boost patient care in the country. It would also increase medical tourism to the country since treatment could be provided in accredited facilities that are at par with those in developed countries but at significantly lower cost. Further, it has been suggested that all relevant RD groups could collaborate to start a gene therapy consortium wherein facilities could be shared, and research for developing gene and cell therapies could be started.

The 14 PGs referred to above believe that there is a need to raise funds for RD research in India. However the others feel that in a resource-limited setting resources should be channeled to meet the immediate needs of patients.

### Achievements

The PGs have had many important achievements which have helped to improve RD patient care and overall RD scenario in India. However, it is not possible to list the enormous contributions of each organization. Therefore we have discussed some of the most important achievements below. Through its extraordinary efforts, the RD community has been able to garner support from both government and industry (Additional file 2). The most important achievement was the release of the National Policy for Treatment of Rare diseases (NPTRD) by the Ministry of Health and Family Welfare, Government of India in May 2017. Unfortunately, this policy was put in abeyance in late 2018. Some state governments have also provided support through various initiatives such as prenatal diagnostics, newborn screening, diagnosis or treatment. The Rights of Persons with Disabilities Act, 2016 covers a wide range of disabilities, including 10 of the 15 RDs in this study (Additional file 2).

ORDI, in collaboration with the Indira Gandhi Institute for Child Health, Bangalore, has helped set up the country’s first Centre of Excellence (CoE) for rare diseases, which coordinates a wide range of services, and provides multidisciplinary expertise and specialized care to patients. This center serves as a hub for Karnataka and aims to tie up with all PHCs across the state to document patient needs and collect patient information. This data will then be used to arrange for consultations with specialists either within the country or abroad, and will also provide key information to research centers and other stakeholders. ORDI is now pitching to replicate this hub–and–spoke model across the country. All the state hubs will be linked to a central CoE. ORDI has been organizing a major public awareness campaign ORDI Race for 7 (www.racefor7.com) which has scaled nationally to 11 cities in India and even extended to 4 cities in US (www.racefor7usa.com). The 7 km walk/run engages the general public in large numbers across the country, includes celebrities who flag off the race, and is widely covered in the media. CHET has developed the Open Platform for Rare Diseases, or OPFORD, a digital support and online resource center for patients, caregivers, and clinicians. It aims to provide information and support for all stakeholders and has been highly successful in raising awareness among the general public using various platforms, including social media.

Following advocacy by HFI, many government hospitals now provide free hemophilia care. Further, the import duty on Anti Hemophilia Factor (AHF) has been waived. Starting from no procurement, over a 10-year period, state health authorities increased their annual purchase of AHF to 200 million IUs in 2017. In the year 2000 HFI received Humanitarian Aid from the World Federation of Hemophilia (WFH). Through this aid, 320 corrective surgeries and 130 minor surgeries have been carried out on hemophilia patients and 35 Immune Tolerance Induction therapies have been carried out at Comprehensive Hemophilia Care Centers. Further, WFH has helped to supply 108 million IUs of AHF to hemophiliacs, for free, for 5 years starting in 2015. IPSPI has also convinced some state governments to provide IVIG treatment to PID patients for free.

Some of the PGs have collaborated with companies to make RD drugs and other products accessible to and affordable for patients. Through the efforts of organizations such as ORDI and LSDSS, enzyme replacement therapies (ERT) for lysosomal disorders have become locally available. To make these prohibitively priced therapies accessible to patients, these PGs have arranged free ERTs for some patients through various initiatives such as the Charitable Access Programs of Sanofi-Genzyme and Shire; the Employee State Insurance Corporation or direct financing of the patient from the Central government or a State government. In 2017, 240 LSD patients received free ERT [35]. Genzyme also provides free Dried Blood Spot (DBS) kits for the diagnosis of Pompe’s disease. For one year, Sun Pharmaceuticals provided iron chelating drugs free-of-charge to 50 thalassemics. TSCS has been supported by various companies to establish a blood bank. This PG provides free blood transfusions and iron chelating drugs which are made possible by individual donors and by blood bank revenues. In an instance of support for an individual patient, in late 2016 a parent crowd-sourced Rs. 12 million for BMT for his six-year-old daughter with LSD.

The PGs have also helped to raise public awareness about RDs by organizing various race, marathon’s and other such public events. ORDI has been organizing a major public awareness campaign ORDI Racefor7 (www.racefor7.com) which has scaled nationally to 11 cities in India and even extended to 4 cities in US (www.racefor7usa.com). The 7 Km walk/run engages general public in large numbers across the country with massive coverage in mass media including celebrities that flag off the race. Similarly, DART has organized the ‘Master Muscle Marathon’ in 2017. Amongst other things, I-ORD and many other PGs have organized a long list of national and international conferences in Delhi, Hyderabad, Lucknow, and so on, across India. All these have helped to raise awareness of RDs in the medical, political, corporate and bureaucrat communities. I-ORD has also been involved with Indian organizations such as the Pharmaceuticals Export Promotion Council of India.

I-ORD and ORDI are both members of several international bodies, such as the Asia Pacific Alliance of Rare Disease Organizations, the European Organization for Rare Diseases (EURODIS), the International Conference on Rare Diseases and Orphan Drugs (ICORD), the National Organization for Rare Disorders (NORD), Rare Diseases International and the United Nation’s NGO Committee for Rare Diseases, and have raised the voice of the Indian RD community at these fora. Other DSGs are also associated with their international counterparts, and this helps them to keep abreast of the latest treatment and management options (Additional file 2).

### Recommendations

Based on their progress to date, and their understanding of the community’s immediate and long-term needs, each PG has a wish list (listed in Additional file 2). Some of the most important recommendations which were common to most of the PGs in our study are summarized below:

i). Every state should implement all the recommendations of the NPTRD promptly and properly.
ii). Medical associations must raise awareness of RDs in the medical community and the general public. It is important to organize Continuing Medical Education (CME) sessions to make doctors aware of various RDs and the latest treatment and management options.
iii). Prenatal and newborn screening should be included in regular medical care.
iv). Both the government and private insurers should establish special insurance programs to cover the treatment and long-term management of RD patients.
v). Schools should be more accommodative of patient needs.
vi). Companies should be motivated to fund RD research and treatment as part of their Corporate Social Responsibility activities.
vii). The Rights of Persons with Disabilities Act, 2016 should be strengthened and properly implemented to safeguard the rights of disabled persons. The Constitution of India, which prohibits the state from discriminating against any citizen on grounds such as caste or gender, should make discrimination on the basis of a disability a punishable offence.
viii). The government should pass ‘Compassionate use’ and Right-to-Try laws so that patients can access molecules not yet approved as drugs. Just as these laws have enabled American patients to access such experimental molecules, which are still in clinical trials, the Indian government should allow the import or development of these therapies on compassionate grounds.
ix). Government should encourage research and drug development of RDs by enacting policies similar to that of Orphan Drug Act in the US.
x). Prevalence studies of RDs should be initiated and the national registry for RDs should become functional.

## Discussion

As mentioned above, the US was a pioneer in efforts to address the needs of RD patients. This was followed by movements in the EU and in Japan. Although Indian efforts to strengthen the ecosystem for RD patients have been much later than those of these three countries, they are contemporaneous with those of many other countries [15]. Canada, for instance, has not yet enacted orphan drug legislation, despite years of pressure from local RD support groups [14]. This study provides an insight into the efforts and achievements of various Indian PGs.

**Fig. 1:**
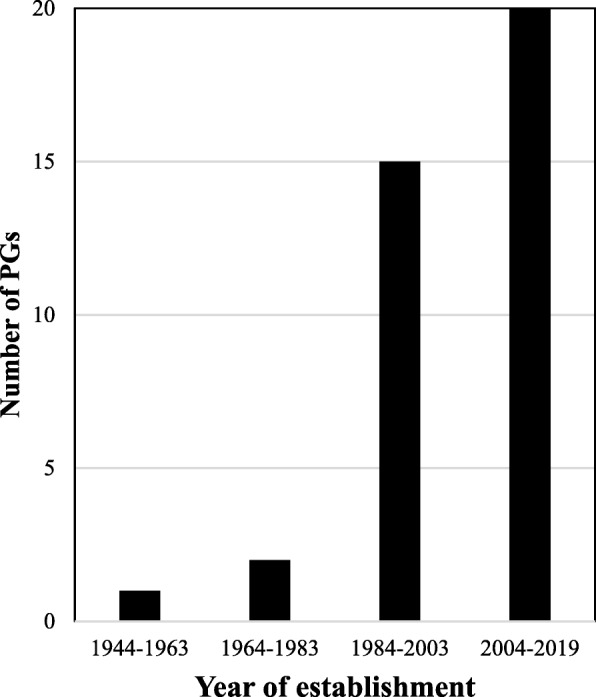
The history of establishment of RD PGs in India. Only PGs with known dates of establishment are included

Most PGs started by providing support services and a platform for members to share their experiences, but their work has evolved over time. DSGs such as AEHF, IRSF and SI are primarily focused on patient care and support. These PGs enable patients or their caregivers to mentor others on various aspects of disease management, which is in tune with the worldwide trend of PGs empowering patients and caregivers with reliable information. Orphanet lists more than 40,000 web-based resources for more than 6900 RDs [36]. The Indian UOs and OONS, all have lists of DSGs on their websites. FRRDD lists 45 PGs on its website and this is by far the longest list in India. The oldest PG was established in 1944, but most have been started in the last 20 years (Fig. 1). Almost all the PGs are located in major cities, with 24 of them headquartered in Bangalore, Delhi or Mumbai. 10 of the PGs of this study are on the FRRDD list. Further, each DSG has its own website, which provides information related to disease management, the latest treatment options available globally, useful home remedies and contact information of clinicians and hospitals.

Globally, many PGs are unable to undertake advocacy due to a lack of funds and manpower, and several Indian PGs have the same constraint [29]. However, in Europe and the US most PGs are linked to a major network such as EURODIS, the Genetic Alliance, Global Genes, ICORD or NORD. These large alliances give patients a strong voice both nationally and internationally [37]. Such advocacy has had a long-term impact on the political agenda, and RDs now consistently receive the attention of policy makers, including in budgetary allocations [38]. In India too, such UOs have emerged, and their activities have resulted in a significantly increased awareness of RDs and patient needs. They have also lobbied the government for policy changes. As a consequence, the central government has planned to initiate support through policies such as NPTRD, and one state government is considering a draft document entitled The Karnataka Rare Disease and Orphan Drugs Policy [39]. The NPTRD mentions that a corpus fund will be created for RD patients each year, and therefore there is hope of sustained financial support by the government. In fact, the NPTRD is a comprehensive document, and the interviewees made suggestions which overlapped 18 of the 23 points in the NPTRD. The five other points made in the NPTRD were (i) the need for an Inter-ministerial Committee to coordinate the RD activities across departments and ministries; (ii) the need for Central- and State-level committees to manage the funds allocated for RDs, including developing criteria of eligibility, funding per patient and so on; (iii) the need for the creation of a corpus fund, both at the Central- and State-level to fund RD patients’ treatment; (iv) the need to remove import duty on certain RD products and (v) the need for standardization of diagnosis, management and treatment protocols, which would need to be periodically revised. These points were not explicitly part of the questionnaire, and that may be the reason the interviewees did not make these suggestions. However, the NPTRD was put in abeyance in November 2018 [40]. This lead to protests from the entire RD community in the country, following whichORDI filed a public interest litigation (PIL) with the Supreme Court to reinstate it. In February 2019, there was a Delhi High Court hearing on this subject. As a result, the government committed to release a revised policy within nine months [41].

One of the most important commitments in the NPTRD is the creation of the Indian Rare Disease Registry (http://bmi.icmr.org.in/irdr/index.php) designed by the Indian Council of Medical Research (ICMR). This has been welcomed by all PGs, but although launched in April 2017 [11] the registry is not yet functional. One way to speed up the process would be for the national registry to compile data from existing registries, or coordinate with the DSGs which could maintain their respective registries. Further, prevalence studies should be undertaken keeping in mind international experience and standards [42]. Such studies would also help to come up with a definition of RDs suited to India, another important goal of NPTRD. Through this policy, the government also committed to create a portal that would list details of the corpus fund, and the mechanism for patients to apply for funding. However, the interface is not very user-friendly. Also, staff in the PHCs need to be trained to guide people to this website and help them apply for funds. Presumably, this portal is also currently non-functional until the NPTRD is reinstated.

As mentioned earlier, PG advocacy in India has also led to support from other stake-holders through initiatives such as the ‘Diet for Life’ program of FSSAI, subsidized IVIG, free AHF, public awareness campaigns such as Race for 7 and the setting up of CoEs for RDs. PGs’ efforts have enabled beta thalassemia patients to access transfusion, iron chelation therapy and BMT, all of which have significantly improved the quality of life of these patients in recent years [43]. Further, the PGs have played an active role in bringing some orphan drugs to the country for the first time, and making them affordable. However, multifaceted strategies are needed to make all drugs for RDs accessible even in the long-run. A policy to encourage the local manufacturing of generic drugs for RDs, possibly by public sector companies, would help to make the large number of off-patent and repurposed orphan drugs readily available and more affordable.

In a large and diverse country such as India, the CoEs for RDs need to develop networks to overcome geographical and language constraints, and cater to more patients [44, 45]. Most importantly, and as for the health of the entire population, the government should implement measures that address regional disparities in healthcare [46, 47]. Furthermore, due to diverse sociocultural issues, particular communities may need tailored healthcare guidelines. Illustratively, consanguinity is accepted in certain communities although it is strictly prohibited in others, and therefore the guidelines for the former groups would need to take this into account [48, 49].

It is essential that research for the development of new diagnostic, treatment and management options be supported. As seen in other countries, RD research in India in areas such as epidemiology, disease etiology, genetics, supportive technologies and so on has largely been driven by the PGs [32, 33, 50–52]. However, these efforts are still in their infancy and more organizations need to draw inspiration from their global counterparts and be involved in research [53]. As suggested by others, causative genes need to be discovered, the natural history of each disease needs to be understood, suitable animal and cellular models need to be created, and clinical trials of promising therapeutic approaches need to be conducted [54]. Indian PGs are now visible at international fora and this will encourage international collaborations for advocacy and research, which are important to improve the RD ecosystem in any country [54].

Despite the efforts of the PGs, and some initiatives by the government, there are many unmet needs in key areas such as awareness, newborn screening and medical insurance. Although newborn screening for 30 conditions was introduced through a national program, the Rashtriya Bal Swasthya Karyakram, in 2013, less than 1% of Indian newborns are screened. This is in contrast to China, where 87% of the babies are screened for two or more conditions at birth [55]. In many developed countries, despite the ready availability of good diagnostic facilities, it can take over seven years on average to diagnose an RD patient [14]. Awareness among doctors is the key to an early diagnosis, which will lead to timely management of the disease, and this holds true for all countries. Further, the high cost of RD treatment is of particular concern in India, where over 70% of healthcare expenditure is out-of-pocket [56]. There are only a few programs such as the Rashtriya Swasthya Bima Yojna and the recently announced Ayushman Bharat initiative which cover some of the healthcare needs of underprivileged patients. However, the initiatives may be poorly implemented. This is in contrast to most developed nations where most citizens are covered by private or public health insurance or receive free medical care [57].

## Conclusions

Most of the PGs were started by people directly affected by an RD. Lack of awareness among health providers, the high cost of treatment, the lack of health insurance and, indirectly, the absence of prevalence data, are the biggest challenges that patients face. This study also highlights the recommendations made by each PG which should catch the attention of policy makers. Looking at the achievements of the Indian PGs already, it is certain that if they were more actively involved in policy formation and implementation, it would significantly improve the RD ecosystem of the country. The government should institutionalize PG participation in formal decision making, as has been done in the Netherlands [58]. It is our hope that studies such as this one will inspire patients with other RDs, which are not yet represented by a PG, to raise their voices, or join forces with a related DSG or with a UO.

## Additional files

Additional file 1: Questionnaire used for the interviews. (DOCX 19 kb)

Additional file 2: **a**) Details of each PG**.** Name, legal status, international partners, regional chapters, whether first PG in India, patient registry, number of patients associated with the PG and website. **b**) RD management. Name of PG; PGs’ views on alternative treatment options; globally available modes of management or treatment not yet available in India; whether prenatal diagnosis is possible; counselling provided to patients by the PGs; research by the PGs, directly or in collaboration; and whether RD is covered by the Rights of Persons with Disabilities Act, 2016. **c**) Challenges faced by the PGs. Challenges faced by patients in the diagnosis, treatment and management of the disease or condition. **d**) Support received by PGs from various sources. Support was received from the following sources, largely within the country: central government; local authorities or state government; public (crowd funding); industry; healthcare providers; philanthropic organizations; company CSR funds; local organizations or NGOs. **e**) Recommendations made by the PGs to various stake holders. **f**) Interviewee story. Each interviewee had a reason to join or start a PG. These stories are captured here in brief. (XLSX 38 kb)

## Abbreviations

AEHF: Asha Ek Hope Foundation
AHF: Anti hemophilia factor
ASHA: Accredited Social Health Activists
BMT: Bone marrow transplantation
CHET: Centre for Health Ecologies and Technology
CME: Continuing medical education
CoE: Centre of excellence
CP: Cerebral palsy
DART: Dystrophy Annihilation Research Trust
DBS: Dried blood spot
DS: Down syndrome
DSFI: Down Syndrome Federation of India
DSG: Disease specific group
ERT: Enzyme replacement therapies
EU: European Union
EURODIS: European Organisation for Rare Diseases
FRRDD: Foundation for Research on Rare Diseases and Disorders
FSSAI: Food Safety and Standards Authority of India
FXS-I: Fragile X Society – India
GM: GNE myopathy
HFI: Hemophilia Federation (India)
ICMR: Indian Council of Medical Research
ICORD: International Conference on Rare Diseases and Orphan Drugs
IEM: Inborn error of metabolism
IICP: Indian Institute of Cerebral Palsy
Iksha: Iksha Foundation
I-ORD: Indian Organization for Rare Diseases
IPSPI: Indian Patients Society for Primary Immunodeficiency
IRSF: Indian Rett Syndrome Foundation
IVIG: Intravenous immunoglobulin
LSD: Lysosomal storage disorder
LSDSS: Lysosomal Storage Disorder Support Society
MERD India: Metabolic Errors and Rare Disease Organization of India
MSSI: Multiple Sclerosis Society of India
NORD: National Organization for Rare Disorders
NPTRD: National Policy for Treatment of Rare diseases
OI: Osteogenesis imperfecta
OPD: Outpatient department
OPFORD: Open Platform for Rare Diseases
ORDI: Organizations for Rare Diseases India
PG: Patient group
PHC: Primary Health Centre
PID: Primary immune deficiency
RD: Rare disease
SI: Sjögren’s India
SS: Sjögren’s syndrome
TSCS: Thalassemia and Sickle Cell Society
UO: Umbrella organization
US: United States of America
WWGM: World Without GNE Myopathy
