# Supplementary material for: The role of patient organizations in the rare disease ecosystem in India: an interview based study
Source: Orphanet J Rare Dis. 2019 May 29;14:117. doi: 10.1186/s13023-019-1093-6 (PMC6542017; doi:10.1186/s13023-019-1093-6)
Supplement: Supplementary file 1 — Questionnaire used for the interviews. (DOCX 19 kb) [file 13023_2019_1093_MOESM1_ESM.docx]

**Additional file 1: Questionnaire used for the interviews**

Interviewee:

Organization:

Interviewer : Dr. Mohua Chakraborty Choudhury

Date :

Time:

Mode:

**Questionnaire:**

**Advocacy group details**

1. How did the interviewee get involved with a rare disease?
2. When:
3. Relation with patient (if applicable):
4. Which rare diseases does this group cover?
5. How did the patient advocacy group start (or became a part of an international body)? What was the driving force behind such an initiative?
6. How did you get connected to other patients to form the patient advocacy group?
7. Has the group registered as a society or trust or company (and if so, as a for-profit or not-for-profit company) or as the Indian chapter of an international organization?
8. Number of patients associated with the group:
9. How do you enrol new patients:
10. Are the advocacy groups trying to identify unreported cases, and if so how ?
11. Do you enquire about the family lineage of patients for carriers and those affected by the disease?

**Disease and patient [this section will apply only to cases where the interviewee is the patient/is related to a patient]**

1. How did the disease get diagnosed, and how much time was spent to get the correct diagnosis? How much did the whole process cost?
2. When the patient was diagnosed, what was available then in terms of:
   1. Diagnosis:
   2. Treatment:
   3. Management:
3. How has the family coped with the situation? Have they reached out to any professional counsellors?
4. Has the patient been able to participate in mainstream schooling/ a professional environment? What support did he/she get ? What kinds of challenges did he/she face?
5. Have you tried any alternate treatment option like ayurvedic/homeopathy?
6. What was the age of disease onset?
7. Any influence of external factors like food, weather or physical activities in the management and manifestation of the disease?
8. What are the complications of the disease? Do they arise from the disease or from medication?
9. Any generic drugs used for treatment?
10. What are the various mode of treatment/management available globally and how many of them are available in India, and at what cost?
11. Is your organization involved with foreign organisations in R&D (including clinical trials) or otherwise?
12. What is known about the prevalence of the disease in India?
13. Is prenatal diagnosis possible?
14. Is the disease endemic to a particular region or ethnic group in India?

**Challenges**

1. What were the challenges for the patient in terms of
   1. Diagnosis
   2. Treatment
   3. Disease management
   4. Schooling
2. What were the challenges you faced in starting the advocacy group?
   1. Learning how to do advocacy work
   2. Reaching out to more patients
   3. Involving clinicians
   4. Financials
   5. Any other

**Support**

1. What help did you get from:
   1. The central government:
   2. The local authority or state government:
   3. The public:
   4. Industry
   5. Health care providers:
   6. Local organization or NGOs

Has any big philanthropic organization helped in any way?

Has any company helped through CSR or in any other way?

Any other source of help?

1. What have been the changes in the environment, in terms of the support available, from the time they started the treatment? And, from the start of the advocacy work?
2. What are your recommendations on policy changes for:
   1. The central government
   2. The state government
   3. The local authorities
   4. Industry
3. How can research help? Have you been associated with any research studies? Any benefits that have accrued to your group or anyone else due to such studies?
4. How can the school and social structure be sensitized? How much of this is happening?
5. Do you have any group activities to strengthen the support group for such patients?
6. What are the organization’s contributions (if any) in the areas of :
   1. disease prevalence
   2. a database or registry
   3. prenatal diagnosis
   4. counselling to caregivers
